# Assessing the functional vulnerability of woody plant communities within a large scale tropical rainforest dynamics plot

**DOI:** 10.3389/fpls.2024.1372122

**Published:** 2024-04-17

**Authors:** Cheng Sun, Jie Yao, Han Xu, Chaofan Zhou, Runguo Zang

**Affiliations:** ^1^ Key Laboratory of Forest Ecology and Environment of National Forestry and Grassland Administration, Ecology and Nature Conservation Institute, Chinese Academy of Forestry, Beijing, China; ^2^ Co-Innovation Center for Sustainable Forestry in Southern China, Nanjing Forestry University, Nanjing, China; ^3^ Research Institute of Tropical Forestry, Chinese Academy of Forestry, Guangzhou, China

**Keywords:** tropical rainforest, functional traits, functional redundancy, species trait distinctiveness, functional vulnerability, environmental factors

## Abstract

**Introduction:**

Tropical forests are characterized by intricate mosaics of species-rich and structurally complex forest communities. Evaluating the functional vulnerability of distinct community patches is of significant importance in establishing conservation priorities within tropical forests. However, previous assessments of functional vulnerability in tropical forests have often focused solely on isolated factors or individual disturbance events, with limited consideration for a broad spectrum of disturbances and the responses of diverse species.

**Methods:**

We assessed the functional vulnerability of woody plant communities in a 60-ha dynamic plot within a tropical montane rainforest by conducting in silico simulations of a wide range disturbances. These simulations combined plant functional traits and community properties, including the distribution of functional redundancy across the entire trait space, the distribution of abundance across species, and the relationship between species trait distinctiveness and species abundance. We also investigated the spatial distribution patterns of functional vulnerability and their scale effects, and employed a spatial autoregressive model to examine the relationships between both biotic and abiotic factors and functional vulnerability at different scales.

**Results:**

The functional vulnerability of tropical montane rainforest woody plant communities was generally high (the functional vulnerability of observed communities was very close to that of the most vulnerable virtual community, with a value of 72.41% on average at the 20m×20m quadrat scale), and they exhibited significant spatial heterogeneity. Functional vulnerability decreased with increasing spatial scale and the influence of both biotic and abiotic factors on functional vulnerability was regulated by spatial scale, with soil properties playing a dominant role.

**Discussion:**

Our study provides new specific insights into the comprehensive assessment of functional vulnerability in the tropical rainforest. We highlighted that functional vulnerabilities of woody plant communities and their sensitivity to environmental factors varied significantly within and across spatial scales in the tropical rainforest landscape. Preserving and maintaining the functionality of tropical ecosystems should take into consideration the variations in functional vulnerability among different plant communities and their sensitivity to environmental factors.

## 1 Introduction

Biodiversity plays a pivotal role in shaping ecosystem functioning (Hooper et al., 2005). Understanding how biodiversity regulates ecosystem functions is the foundation for biodiversity conservation and ecosystem management practices (Tilman et al., 2014). In ecosystems, species with diverse trait syndromes may perform various functions, and the loss of biodiversity will directly influence ecosystem functioning (Lavorel and Garnier, 2002). To understand the functionality of an ecosystem, one must consider not only the quantity of species but also the functional characteristics of each species (Hooper et al., 2005). Functional traits are defined as morpho-physio-phenological traits that influence adaptation through their effects on individual performance, including growth, survival, and reproduction (Violle et al., 2007; Schmitt et al., 2020). These traits play a pivotal role in how species respond to environmental changes and influence ecological processes (Lohbeck et al., 2012). Functional diversity (FD) refers to the distribution of species and their abundances in the functional trait space of a given community (Mouillot et al., 2013). FD integrates both species and ecosystem characteristics, facilitating the examination of a wide range of ecosystem functions by considering different combinations of traits. This has been instrumental in revealing the underlying mechanisms that govern the interplay between biodiversity and ecosystem functionality (Mason et al., 2013).

Ecosystems are continually exposed to diverse external pressures and disturbances, and the ability to sustain their functional diversity is paramount for the health and stability of ecosystems (Mason et al., 2013; Suding et al., 2015). Functional vulnerability refers to the degree to which FD is likely to change when exposed to external pressures and disturbances (Turner et al., 2003). The core focus of functional vulnerability is whether ecosystems can preserve their stability when exposed to diverse external pressures and disturbances, as well as their capacity for resistance and recovery in the presence of such disruptions (Arnaud et al., 2022). Assessing functional vulnerability is essential for maintaining ecosystem health, conserving biodiversity, and rationalizing ecosystem management and conservation efforts (Comte and Olden, 2017; Liu et al., 2023). Over the years, there has been significant focus on evaluating the functional vulnerability of ecosystems, with researchers consistently emphasizing the importance of quantifying and exploring this theme (Turner et al., 2003; Mouillot et al., 2013a; Arnaud et al., 2022). Early assessment methods related to functional vulnerability primarily emphasized the measurement and monitoring of ecosystem functions, such as material cycling, productivity, and energy flow (Pimm, 1984). Subsequently, with the introduction of the concept of functional diversity, researchers gradually shifted their focus from solely species diversity to the study of species functional traits and functional diversity, thus enhancing our understanding of ecosystem functionality (Díaz and Cabido, 2001). Currently, the progress in quantitative ecology and ecosystem modeling enables researchers to simulate how ecosystems respond to different pressures and disturbance conditions, which facilitates the evaluation of functional vulnerability (Smith et al., 2014; Shiogama et al., 2016; Forzieri et al., 2021; Trew et al., 2021).

Despite the progress made in ecosystem vulnerability assessment, the majority of studies have been concentrated on specific disturbances, such as wildfires (Armenteras et al., 2021), droughts (Chen et al., 2022) or logging (Smith et al., 2023). Indeed, the complexity of biological responses and the exposure to multiple disturbances require us to take into account a wide range of uncertain disturbance types when evaluating the functional vulnerability of ecosystems (Staudt et al., 2013; Arnaud et al., 2022). The method for assessing functional vulnerability, as proposed by Arnaud et al. (2022), integrates community species’ functional distinctiveness, species abundance distribution, and the distribution of functional redundancy across the entire trait space. This method enables us to measure how susceptible ecosystem functionality is to various disturbances by conducting in silico simulations of a wide range of disturbances. Compared to alternative approaches for measuring functional vulnerability, this method enables users to comprehensively consider a system’s vulnerability, rather than focusing solely on the species level or evaluating a particular disturbance. Indeed, considering the complexity of biological interactions at the community scale and the lack of inherent knowledge about various pressures, this is a necessary step towards achieving effective ecosystem management (Bartomeus and Godoy, 2018).

Forest communities play a vital role as reservoirs of Earth’s biodiversity, serving as a primary biological factor that ultimately influences ecosystem functionality (Loreau and Hector, 2001). Species functional traits and ecological strategies often exhibit significant differences across different community patches within a larger area (Lavorel and Garnier, 2002; Díaz et al., 2007; Mouillot et al., 2013). Communities with high functional vulnerability imply low insurance effects and diminished resilience after disturbances (Mouillot et al., 2014). Therefore, understanding the spatial distribution pattern of functional vulnerability within forest communities is crucial for effectively identifying vulnerable areas and formulating strategies for intervention and maintenance management. The spatial distribution pattern of functional vulnerability is closely related to habitat heterogeneity, which reflects the variations in plant communities’ responses to various disturbances across different habitat types (Thuiller et al., 2011). Both biotic and abiotic factors together drive the changes in functional vulnerability (Mouillot et al., 2013). For instance, soil nutrients play a pivotal role in shaping the composition of communities and the diversity of functions within various types of habitats (Peguero et al., 2023), thereby impacting the manifestation of functional vulnerability in different communities. The complexity of forest stand structure contributes to the enhancement of community functional diversity, thereby augmenting resistance to disturbances (Lian et al., 2022). Furthermore, understanding the variation in functional vulnerability across various spatial scales is crucial for identifying how different ecological processes impact the stability of community function (Chase and Knight, 2013; Kadowaki et al., 2018). Given the intrinsic connection between spatial scale and ecosystem functioning, ecological drivers and processes that are dependent on species and scale interact with each other. These interactions can lead to varied effects at different scales (Lang et al., 2009; Bastos et al., 2016).

Tropical rainforest is the terrestrial ecosystem with the highest biodiversity on Earth (Steffan-Dewenter et al., 2007). Its rich array of species plays a crucial role in maintaining the global ecological balance and resulting human well-being (Freda, 2022). However, widespread ecological threats stemming from human activities are placing the tropical rainforest at a significant risk of biodiversity loss and degradation of ecosystem function (Malhi et al., 2020). Uncertainty about future environmental change makes planning for tropical rainforest management exceptionally challenging (Lewis and Maslin, 2015; Barlow et al., 2016; Swamy et al., 2018; Tao et al., 2022). Identifying the functional vulnerability of tropical rainforest communities and their driving factors is urgently needed. This is essential for developing more effective conservation strategies and sustainable management plans aimed at preserving tropical rainforests biodiversity and ecosystem functionality in tropical rainforests (Naeem et al., 2012). Nonetheless, our comprehension of the distribution patterns and driving factors behind functional vulnerability in tropical rainforest remains limited. This study conducted a functional vulnerability assessment of woody plant community patches at various spatial scales (20 m×20 m, 40 m×40 m, 60 m×60 m, 80 m×80 m and 100 m×100 m) within a 60-ha heterogeneous tropical rainforest landscape on Hainan Island, China. We performed an analysis of the distribution pattern of functional vulnerability, investigating trends in functional vulnerability across different spatial scales. Furthermore, we explored how both biotic and abiotic factors contribute to functional vulnerability and examined whether spatial scale modulates these contributions. Specifically, our primary focus was on addressing the following questions: a) How does the functional vulnerability of tropical rainforest woody plant communities change? What is their spatial distribution pattern? b) To what extent do biotic and abiotic factors influence variations in community functional vulnerability? And is the impact of these factors modulated by spatial scale?

## 2 Materials and methods

### 2.1 Study site

**Figure 1:**
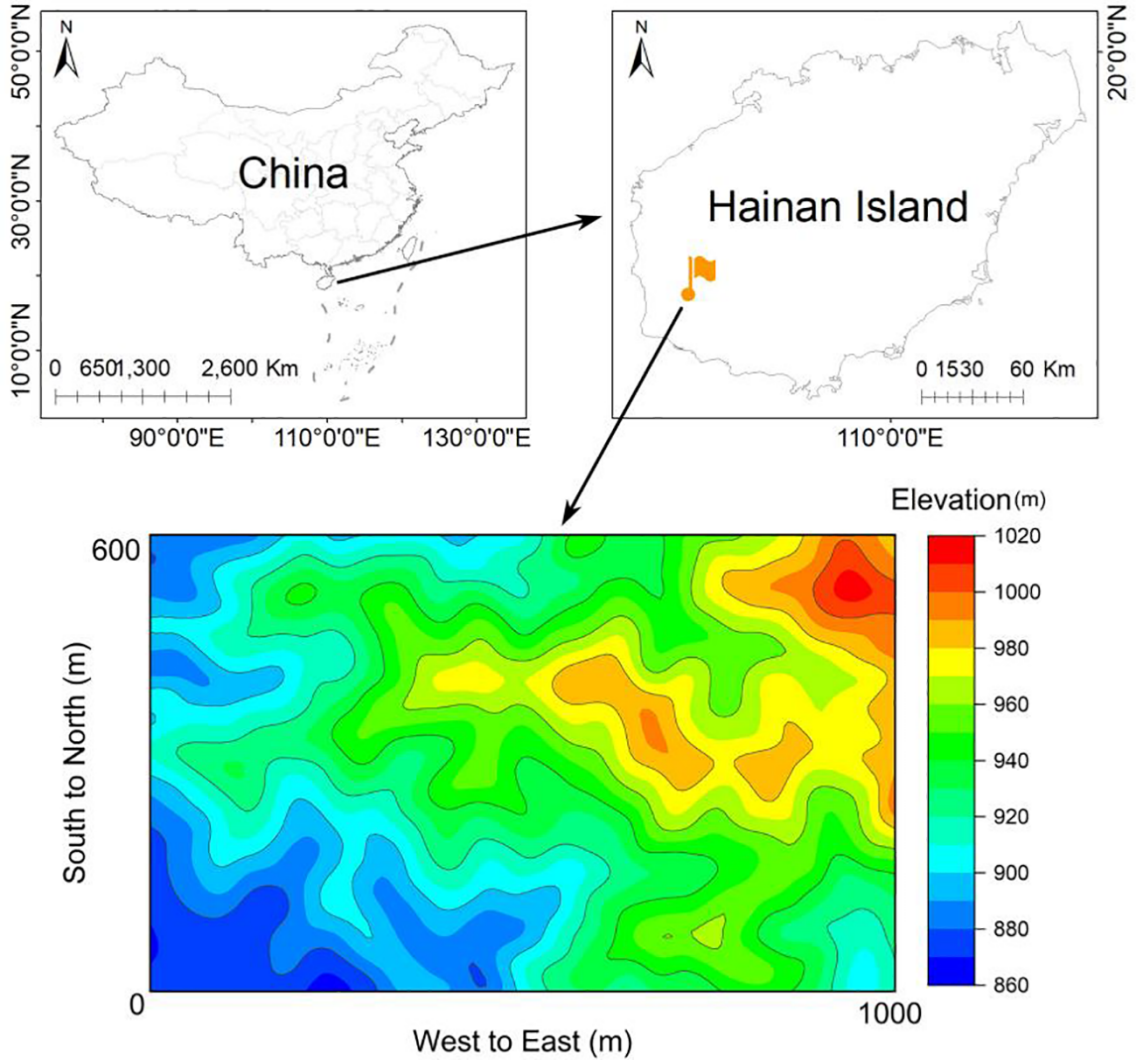
Sketch map of the 60ha FDP in the Jianfengling National Nature Reserve in Hainan Island, China.

This study was carried out in Jianfengling National Nature Reserve in the southwest of Hainan Island, China, with geographical coordinates of (18°23′-18°50′N and 108°36′-109°05′E). A 60-ha FDP has been established in the core area of the Nature Reserve, serving as a typical seasonal moist tropical rainforest landscape (**Figure 1**; **Supplementary Table S1**). In the study area (60-ha FDP), the elevation ranges from 866m to 1016m, the annual average temperature is 24.5°C and the monthly average ranges from 19.4°C to 27.3°C, with a wet season from May to October and a dry season from November to April (Zang et al., 2019). The FDP was divided into 1,500 0.04-ha (20m×20m) subplots, which served as the basic units for investigation and monitoring. Within each subplot, identify and label all species of erect woody plants with a diameter at breast height (DBH)≥1cm, including both trees and shrubs, while measuring their tree height and DBH.

### 2.2 Functional trait measurements

Six plant functional traits (**Table 1**) were measured using standard methods (Pérez-Harguindeguy et al., 2013). These functional traits are significantly important for comprehending plant growth, survival, and their reactions to environmental conditions (Bello et al., 2010; Lohbeck et al., 2012) (**Table 1**). In each subplot (20m×20m), a minimum of 10 individuals per species were sampled (species with fewer than 10 individuals were entirely sampled). For each plant, 5 ~ 10 fully grown leaves were chosen. The method for measuring plant functional traits is consistent with the method used by Zhang and Zang (2021). The functional trait data in this paper are all weighted averages.

**Table 1:** A description of the six measured functional traits.

Traits	Major functional relevance
Leaf dry matter content (LDMC, g/g)	Leaf defense (Wright et al., 2004)
Specific leaf area (SLA, cm^2^/g)	Light interception efficiency (Liu et al., 2022)
Wood density (WD, g/cm^3^)	Efficiency and safety of water transport (Yang et al., 2024)
Leaf nitrogen concentration (LNC, mg/g)	Photosynthetic capacity (Han et al., 2005)
Leaf phosphorus concentration (LPC, mg/g)	Growth and photosynthetic capacity (Han et al., 2005)
95% quantile of height for all individuals per species (H_max_, m)	Growth and competition (Kunstler et al., 2016)

### 2.3 Measurement of biotic and abiotic factors

The biotic factors identified for each subplot consist of forest stand structure and species richness (**Table 2**). Stand structure encompasses the standard deviation of tree diameters at breast height, the standard deviation of height, and basal area. We calculated the standard deviation of tree diameters at breast height, standard deviation of height, basal area and species richness in each quadrat at 20 m×20 m、40 m×40 m、60 m×60 m、80 m×80 and 100 m×100 scales. BA was computed using the following formula: BA = (π×DBH^2^)/40000.

**Table 2:** Classification and the respective abbreviations of environmental factors.

Classify	Name	Abbreviation
Soil properties	available nitrogen (mg/kg)	AN
	available phosphorus (mg/kg)	AP
	available potassium (mg/kg)	AK
	pH value	pH
Topographic factors	slope (°)	Slope
	elevation (m)	Elevation
	convexity	Convexity
Stand structure	standard deviation of tree diameters at breast height (cm)	SdDBH
	standard deviation of height (m)	SdH
	stand basal area (m^2^)	BA
Species richness		SR

The abiotic factors encompassed soil properties and topography (**Table 2**). Soil properties assessed for each subplot comprised soil pH, available nitrogen, available phosphorus, and available potassium. The topography included slope, elevation and convexity. Five soil samples from 0 to 20 cm depth were collected at the central point and four corners of each subplot (20 m×20 m), and the five soil samples thoroughly mixed into one Ziploc bag, numbered, then transferred to the laboratory to measure soil properties. The slope was measured using a compass, and the elevation was recorded using a hand-held GPS device; the convexity was then calculated. The values of the soil properties and topography were calculated for larger cells (i.e., 40 m×40 m, 60 m×60 m, 80 m×80 m, and 100 m×100 m) based on the mean values of the 20m×20m cells contained within each larger cell.

### 2.4 Functional vulnerability

We employed the method proposed by Arnaud et al. (2022). to evaluate functional vulnerability. The primary advantage of this method is that it provides an index with absolute values, which can fairly measure the strength of functional vulnerability. Specifically, it was completed by the next three steps:

#### 2.4.1 Building functional entities

Functional entities (FEs) consist of distinct combinations of trait categories (Mouillot et al., 2014). We constructed functional entities by utilizing trait space (Arnaud et al., 2022). Initially, a multi-dimensional trait space was established to depict functional similarity among all species, with the aim of characterizing the functional structure of the observed community. Here, we based on Euclidean pairwise distance to determine the distance matrix between species. Subsequently, we conducted a Principal Coordinates Analysis (PCoA) to transform pairwise species distances into a multi-dimensional space. Second, defining functional entity. Here, we established a two-dimensional trait space based on the first two PCoA axes and placed a regular 20×20 grid cell within it. We considered only the first two PCoA axes because considering additional axes would disperse species into numerous FEs. The boundaries for grid placement are defined by the species located at the extremes of the trait space, including the topmost, bottommost, leftmost, and rightmost species. We defined each 20×20 regular grid cell as a “functional entity” because its position in the trait space serves as a representative proxy for the role of species within the ecosystem (Arnaud et al., 2022) (**Supplementary Figure S1**).

#### 2.4.2 Creating virtual communities

We placed the community’s response to disturbance along the entire spectrum of possibilities, spanning from the ‘least’ to the ‘most’ vulnerable community. We then investigated these two extremes through the virtual communities we constructed. Based on the two-dimensional feature space of the observed community, we generated virtual communities through three community properties: (i) distribution of functional redundancy across the entire trait space, (ii) distribution of abundance across species, (iii) relationship between species trait distinctiveness and species abundance (Arnaud et al., 2022). This leads to a total of 15 virtual communities (**Supplementary Figure S2**).

For functional redundancy and abundances of species, we all adopted three patterns: homogeneous, heterogeneous, and the distribution of the observed community. We obtained both homogeneous and heterogeneous distributions by adjusting the positions of species within the trait space (Arnaud et al., 2022). Concerning the correlation between species trait distinctiveness and abundance, we have chosen to categorize it into two modalities: positive and negative. The species trait distinctiveness (*D_i_*) was calculated as follows (Violle et al., 2017):


(1) $$D_{i}=\frac{\sum_{j=1,j\neq i}^{S}d_{ij}}{S-1}$$

where *S* represents the overall number of species in the species pool, and *d_ij_* denotes the dissimilarity measure between species *i* and *j*. In this research, *D_i_* was standardized, resulting in distinctiveness values ranging from 0 to 1.

#### 2.4.3 Functional vulnerability index

For each observed community and its corresponding virtual communities, we conducted a series of simulated disturbances to quantify their functional vulnerability. At each disturbance, we randomly reduced the total abundance of the community by 5% (the reason for choosing random reduction is because trait-environment relationships and disturbance regimes are unknown, unpredictable, or poorly documented, it is difficult to determine the probability of species being affected based on their sensitivity to specific disturbances). Subsequently, we recalculate the new abundances and determine the number of FEs that still had at least one species present. We applied these simulated disturbances successively until there were no longer any species left within any FEs (**Supplementary Figure S3**). The functional vulnerability index was determined by quantifying the rarefaction curve’s position for the observed community relative to that of both the most and least vulnerable virtual communities (**Supplementary Figure S3**). In conclusion, the community’s functional vulnerability ranges from 0% to 100%, and this calculation was determined in the following manner:


(2) $$Functional\;vulnerability=100\times \left(1-\frac{AUC_{obs}-AUC_{min}}{AUC_{max}-AUC_{min}}\right)$$

where *AUC_max_* represents the area under the rarefaction curve for the least vulnerable community under continuous disturbances, *AUC_min_* represents the area under the rarefaction curve for the most vulnerable community under continuous disturbances, and *AUC_obs_* represents the area under the rarefaction curve for the observed community under continuous disturbances (**Supplementary Figure S3**).

#### 2.4.4 Sensitivity analyses

Sensitivity analysis was performed to examine the potential impact of the number of disturbance series on the functional vulnerability index. The results indicated that the number of disturbance events has a small impact on the functional vulnerability values (the relative standard deviation of functional vulnerability values across multiple disturbance scenarios was always less than 2%) (**Supplementary Figure S4**). To achieve a good balance between computation time and robustness, we performed 2 series of 100 disturbances for each community, resulting in an approximate uncertainty of 0.46% (**Supplementary Figure S4**).

### 2.5 Analysis of the impact of biotic and abiotic factors on functional vulnerability

To discern variations in functional vulnerability across different landform types, we used the Fuzzy C-Means algorithm to collaboratively cluster topography factors at the quadrat of 20m×20m (elevation, convexity, and slope, **Supplementary Figure S5**), classifying the study site into four habitat types: depression, gentle slope, steep slope, and hilltop. We used Analysis of Variance (ANOVA) to test the differences in community functional vulnerability among different habitat types. To estimate the relative importance of environmental predictors including soil, topography, and stand structure for functional vulnerability in communities, we conducted the following analyses. First, the variance inflation factor (VIF) was used to test the multicollinearity of environmental predictors using the R package *car*; a VIF > 10 indicated excessive collinearity (Montgomery et al., 2021), and all of our environmental predictors passed the test (**Supplementary Figure S6**). Then, taking into account spatial autocorrelation, we applied a spatial error model (one type of spatial autoregressive model) to explain the contribution of environmental factors to community functional vulnerability (see **Supplementary Material** and **Supplementary Table S2**). Before that, we utilized the Z-score method to standardize the analytical data, effectively eliminating dimensional disparities among various datasets and thereby improving data accuracy. A spatial weight matrix was constructed using the “queen contiguity” method (**Supplementary Figure S7**) to determine the spatial structure or relationships between adjacent observations. All of the statistical analyses were performed in R 4.2.1 (R Core Team, 2021). The Fuzzy C-Means (FCM) algorithm for collaborative clustering was performed using *hclust()* function in the *Stats* R package (Lovelace et al., 2019). The analysis of spatial error model was performed using *errorsarlm()* function in the *Spdep* R package (Bivand, 2022).

## 3 Results

### 3.1 Spatial distribution patterns of functional vulnerability in woody plant communities within tropical forest

**Figure 2:**
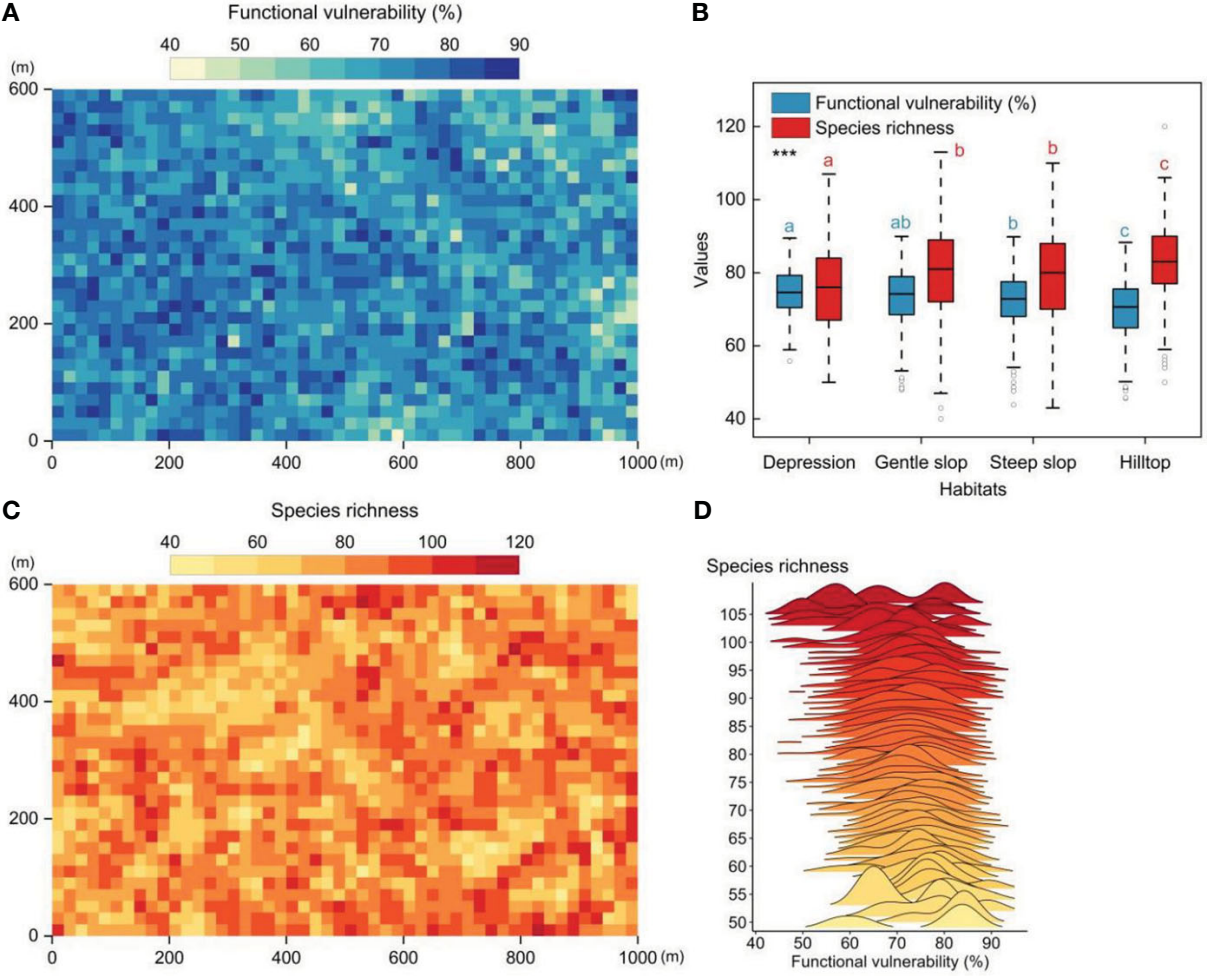
The spatial patterns of functional vulnerability and species richness in tropical mountain rainforest (20m×20m quadrat scale). **(A)** The spatial patterns of functional vulnerability in the 60-ha FDP. **(B)** Functional vulnerability and species richness in four different habitats. **(C)** The spatial patterns of species richness patterns in the 60-ha FDP. **(D)** Distribution of functional vulnerability values along the species richness gradient. Significantly different contrasts (Games-Howell test) are indicated by different lowercase letters. *** indicates the significant level of difference p<0.001.

The 60-ha FDP exhibited distinct functional vulnerability patterns when observed at the 20m×20m quadrat level. These patterns revealed lower vulnerability in the eastern (higher-altitude) regions and higher vulnerability in the western (lower-altitude) regions, exhibiting a patchy distribution (**Figure 2A**). Significant differences in community functional vulnerability and species richness were observed among the four distinct habitats. The overall gradient of community functional vulnerability showed a pattern of depression > gentle slope > steep slope > hilltop (**Figure 2B**). A weak but significant negative correlation between functional vulnerability and species richness (Pearson′s correlation test, *r*=-0.19, *p*<0.01, n=1500) (**Figures 2C, D**).

### 3.2 Functional vulnerability in woody plant community across spatial scales within tropical forest

**Figure 3:**
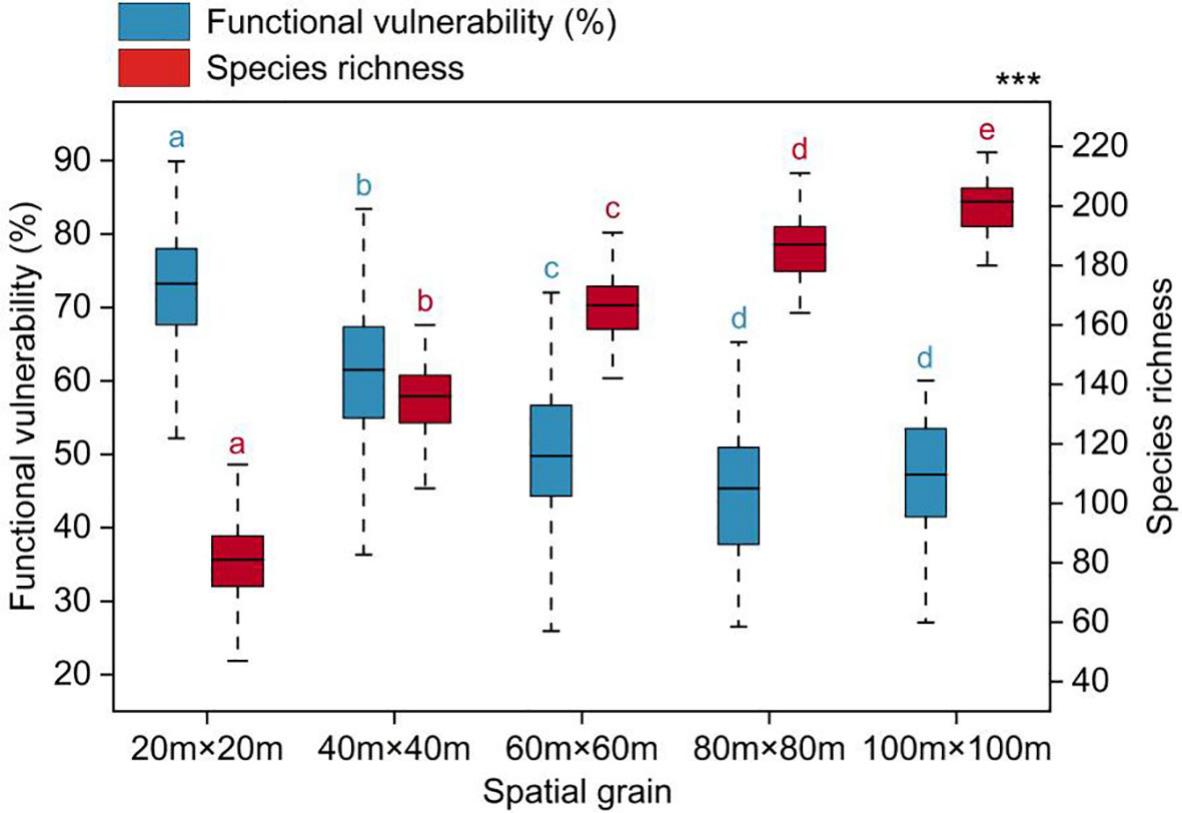
Functional vulnerability varied with spatial grain. Significantly different contrasts (Games-Howell test) are indicated by different lowercase letters. *** indicates the significant level of difference p<0.001.

Functional vulnerability showed significant variation across spatial scales, gradually decreasing and tending to stabilize as the spatial scale increased (**Figure 3**). The average values of functional vulnerability at each spatial scale were as follows: 72.41% (20m×20m), 61.05% (40m×40m), 50.29% (60m×60m), 45.32% (80m×80m), and 46.76% (100m×100m).

### 3.3 Relative contributions of biotic and abiotic factors to the variation of functional vulnerability

**Figure 4:**
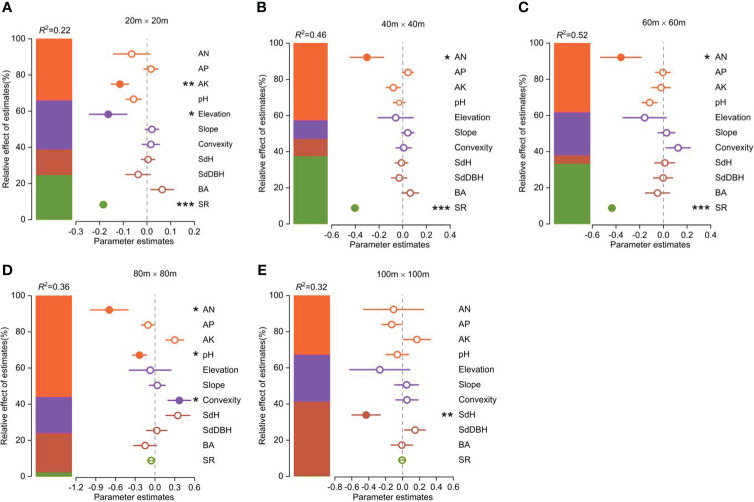
Parameter estimates (standardized regression coefficients) of the fitted model at different spatial scales (the spatial error model showed in **Supplementary Table S1**). Associated 95% confidence intervals and relative importance of each factor, expressed as the percentage of explained variance. Significance level: *p < 0.05; ** *p* < 0.01; *** *p* < 0.001.

The functional vulnerability was strongly influenced by both biotic and abiotic factors (**Figure 4**). Available N, available K, and soil pH were the most important soil factors in determining the degree of functional vulnerability. Elevation and convexity were the most important topography factors in determining the degree of functional vulnerability. The species richness significantly affected the functional vulnerability. SdH was the most important forest stand structure factor influencing the functional vulnerability.

The contributions of these biotic and abiotic factors to the variation in functional vulnerability clearly varied with scale (**Figure 4**). Soil properties had the greatest influence on community functional vulnerability across spatial scales except at the 100m×100m scale. Topographic factors played an important role in shaping the variability of community functional vulnerability at the scales of 20m×20m and 80m×80m. Species richness significantly influenced community functional vulnerability at smaller scales (20m×20m, 40m×40m, 60m×60m), while stand structure played a key role at larger scale (100m×100m).

## 4 Discussion

### 4.1 Functional vulnerability of woody plant communities within tropical rainforest

In this study, we evaluated the functional vulnerability of woody plant communities within a 60-ha tropical montane rainforest dynamic plot to a wide range of potential disturbances. Our results revealed significant spatial heterogeneity in the functional vulnerability of the woody plant community within the study area, characterized by a patchy distribution (**Figure 2A**). The observed distribution pattern of functional vulnerability (**Figure 2A**) primarily reflects the varying response capabilities of distinct woody plant community patches to external disturbances (Hooper et al., 2005). This significant spatial heterogeneity underscores the non-uniformity and complexity of functional diversity within the woody plant community in response to external disturbances. Meanwhile, species richness and habitat conditions often played a crucial role in determining a community’s responsiveness to external disturbances (Hector and Bagchi, 2007). We found species richness and functional vulnerability of communities in different habitats exhibited significant differences, and both displayed opposite trends along the habitat gradient (**Figure 2B**). These findings suggested that habitat variations can substantially influence the community’s response to external disturbances, and species richness had a positive impact on community functional stability.

Species richness had a positive impact on community stability, but it was not ubiquitous (Valencia et al., 2020). On the one hand, we found the distribution patterns of functional vulnerability did not align completely with the distribution patterns of species richness (**Figures 2A, C**). On the other hand, with the continued increase in species richness, it appeared that species richness does not exert a sustained and robust influence on community stability (the existence of a situation where functional vulnerability remains relatively constant or decreases slowly with increasing species richness) (**Figure 2D**). The distribution of species traits within a community may depend on species richness, and as species richness increased, the benefits of species richness may be offset by other community characteristics such as functional redundancy (Arnaud et al., 2022). This is perhaps why there is a weak but significant correlation between species richness and functional vulnerability.

### 4.2 Scale dependence of functional vulnerability

A multitude of ecological processes and their interactions were found to be scale-dependent, suggesting that they could manifest distinct characteristics across various spatial scales (Wiens, 1989). In this study, the functional vulnerability of woody plant communities exhibited significant variations across different spatial scales, with a gradual decline as the spatial scale increased, ultimately reaching a relatively stable state (**Figure 3**). These results indicated an association between functional vulnerability and spatial scale, suggesting that woody plant communities functions may exhibit greater stability and resilience at larger scales within the tropical rainforest (Pickett and Cadenasso, 1995). This is aligned with numerous ecological theories, such as the concepts of ecological compensation and landscape ecology (Naveh and Lieberman, 2013; Bull et al., 2014). One possible explanation for this could be that larger-scale communities typically encompass multiple habitat types, which in turn can support a more diverse species. Different species often play distinct ecological roles, demonstrating strong ecological functional complementarity (Tilman et al., 1997; Loreau and Hector, 2001). Simultaneously, communities at larger-scale harbor more complex ecological interaction networks that encompass interactions among various plant species and interactions between plants and animals, microorganisms, and other organisms (Brooker et al., 2008; Van Der Heijden et al., 2008; Bronstein, 2009). Intricate networks like these have the capacity to alleviate the effects of external disturbances and environmental changes. These change may reflect variations in the complexity and interactions within the ecosystem at different spatial scales (Wiens, 1989), indicating that spatial scale plays an important role in assessing community functional vulnerability.

### 4.3 Correlations of functional vulnerability with biotic and abiotic factors

It was notable that both biotic and abiotic factors that affect functional vulnerability varied significantly (**Figure 4**). An interesting finding from our study is that the overall effect of abiotic factors on functional vulnerability exceeded that of biotic factors. This result suggested that abiotic factors were more important in shaping the functional vulnerability of woody plant communities in tropical forests (**Figure 4**). Earlier studies have shown that the patterns of species composition changes among different forest communities are closely linked to variations in environmental conditions in the study area (Jiang et al., 2016). Functional vulnerability primarily reflects the resilience of a community’s functional diversity in response to external disturbances (Arnaud et al., 2022). Additionally, the assurance in this resilience predominantly arises from the influence of abiotic factors, such as soil nutrients and elevation, which often directly affect community speciation rates and growth status (Vitousek and Howarth, 1991; Körner, 2007; Kraft et al., 2011). As a result, abiotic factors play a more prominent role in shaping both community functional diversity and stability. Therefore, different habitats may shape ecological communities, resulting in distinct relationships between functional vulnerability and local biotic and abiotic factors.

Our results indicated that the effect of environmental variables on functional vulnerability varied with scale. A key finding from our study is that the effect of both biotic and abiotic factors on functional vulnerability did not exhibit complete consistency across spatial scales, highlighting that the influence of both biotic and abiotic factors on functional vulnerability was influenced by the spatial scale. For abiotic factors, soil properties had more influence on functional vulnerability in woody plant communities than topographic factors at different scales. This indicates that higher soil nutrient levels within a community contribute to reducing functional vulnerability, while flat terrain and higher elevation enhanced this effect. Therefore, communities in mid-elevation regions with higher soil nutrient content and flatter terrain tend to have lower functional vulnerability, which aligns with the distribution pattern of functional vulnerability discussed earlier (**Figures 2A, B**). We know that tropical rainforest soils are generally nutrient-poor (Grubb, 1995). In such environments, higher soil nutrient levels within the community can directly promote plant growth and maintain ecosystem functionality (Marschner, 2011; Peguero et al., 2023). Furthermore, compared to low-elevation regions, the habitat conditions in mid-elevation regions are relatively favorable (Körner, 2007; Powers et al., 2015). Meanwhile, the presence of flat terrain is more conducive to the capture of light resources by plants (Grubb, 1995). For biotic factors, functional vulnerability was more susceptible to the influence of species richness at small scales, while it relied more on stand structure regulation at larger scales (**Figure 4**). This might be due to the fact that shifts in species diversity on a smaller spatial scale tend to exhibit greater sensitivity to changes in ecological functions (Hector and Bagchi, 2007). Conversely, at larger spatial scales, stand structure can have an impact on the connectivity and integrity of community habitats, thereby influencing species migration and dispersal. For example, greater community tree height heterogeneity (SdH) may provide additional habitats and refuges (Valladares et al., 2014), potentially playing a more critical role in maintaining the structure and functionality of habitats (Lefsky et al., 2002; Ali, 2019).

### 4.4 Implications for biodiversity conservation and restoration of tropical rainforest landscape

As a consequence of the compounding impacts of deforestation, climate change, and forest degradation, several species in the tropical rainforest are threatened and even endangered (Martin et al., 2011; Roberts et al., 2017). Preserving and maintaining the functionality of tropical forests are crucial for addressing climate change, preserving biodiversity, and promoting sustainable development (Gibson et al., 2011). The Jianfengling National Nature Reserve has undergone extensive logging and rotation processes in the past century. The existing primitive tropical forest has been fragmented into mosaics consisting of secondary forest patches. Further elucidating the functional vulnerability of different plant communities across the heterogeneous forest landscape and their driving factors will contribute to the development of conservation strategies to maintain the functionality of these fragile tropical ecosystems. Our study revealed that the functional vulnerability of woody plant communities in tropical montane rainforest was notably heterogeneity, and it clearly demonstrated scale-dependent effects. Communities in different habitat types displayed significant variations in their levels of functional vulnerability. Furthermore, the mechanisms through which both biotic and abiotic factors influenced functional vulnerability were diverse, with their relative importance depending on the scale. These findings emphasized the intricacy and variety of tropical rainforest ecosystems. Consequently, when it comes to understanding and assessing the functional vulnerability of woody plant communities within tropical montane rainforest, it is necessary to take into account various factors and scales. Additionally, for the preservation of tropical rainforest ecosystem function, it is vital to accurately address the specific ecological system requirements and implement essential protective measures. For instance, determining priorities and optimizing resource allocation for the conservation of tropical rainforest can be based on habitat types. On a smaller scale, there should be an emphasis on conserving species diversity, soil health, and quality to preempt species loss arising from soil erosion and degradation. On a larger scale, endeavors should be geared towards safeguarding forest integrity to ensure the continuity of stand structure and ecosystem.

## 5 Conclusions

This study revealed the distribution patterns of woody plant communities functional vulnerability and its driving factors within a heterogeneous tropical rainforest landscape on Hainan Island, China. Our findings indicated that the functional vulnerability of woody plant communities in a heterogeneous tropical rainforest landscape decreased with increasing spatial scale. Furthermore, the heterogeneity in functional vulnerability is evident among different woody plant communities, with significant differences in community functional vulnerability observed among four habitat types. We highlighted the spatial heterogeneity in functional vulnerability across various spatial scales within tropical rainforest woody plant communities. Additionally, the sensitivity of functional vulnerability to both biotic and abiotic factors varies significantly at different spatial scales. Therefore, in order to preserve and maintain the functionality of diverse plant communities within a heterogeneous tropical forest landscape, it is necessary to consider the variation of functional vulnerability of different plant communities. It is recommended to implement diverse conservation strategies, taking into account the scale-dependent relationships between both biotic and abiotic factors and ecosystems.

## Data availability statement

The original contributions presented in the study are included in the article/**Supplementary Material**. Further inquiries can be directed to the corresponding author.

## Author contributions

CS: Formal analysis, Methodology, Writing – original draft, Software. JY: Formal analysis, Methodology, Software, Writing – original draft. HX: Investigation, Writing – review & editing, Data curation. CZ: Writing – review & editing, Methodology, Software. RZ: Conceptualization, Writing – review & editing, Validation.
