# Supplementary material for: Assessing the functional vulnerability of woody plant communities within a large scale tropical rainforest dynamics plot
Source: Front Plant Sci. 2024 Apr 17;15:1372122. doi: 10.3389/fpls.2024.1372122 (PMC11061514; doi:10.3389/fpls.2024.1372122)
Supplement: Supplementary file 1 [file DataSheet_1.docx]

**Supplementary Material**

Spatial data often lacks independence and exhibits strong spatial autocorrelation. In such cases, a spatial linear regression model can be employed to analyze the relationship between the dependent and independent variables. This approach considers the spatial proximity, accounting for potential similarities between observations in neighboring locations (Hanbat et al., 2023).

In this study, the Moran's Index indicated that all the predictor variables and the response variable exhibited strong spatial autocorrelation. Furthermore, when we applied an ordinary least squares regression model (OLS) to the data, the residuals displayed significant spatial autocorrelation (p <0.001). Therefore, using the ordinary least squares model (OLS) as a benchmark, the environmental variables' relationship with functional vulnerability was modeled using the spatial error model (SEM). The results showed that the spatial error model overperformed the OLS model, not only effectively reduced the spatial dependence, but also improve the model predictions (Table. S2).

The SEM model is expressed as (Anselin and Getis, 1992):

| $Y=\beta X+\xi$ and $\xi=\lambda W\xi+\varepsilon$ | (1) |
| --- | --- |

where *Y* is a vector of the response variable, *X* is a matrix of the explanatory variables, *W* is a spatial weight matrix, *β* is a vector of the regression coefficients, *ɛ* is a vector of model error terms following N(0, σ^2^ I), *λ* is a spatial autoregressive coefficient.

**TABLE S1.** Main tree species in FDP (only the top 20 most abundant species are listed here)

| **Site** | **Main tree species composition** |
| --- | --- |
| **FDP-60ha** | *Prismatomeris tetrandra* (Roxb.) K. Schum., *Cryptocarya chingii* W. C. Cheng.,  *Neolitsea ellipsoidea* C. K. Allen., *Psychotria straminea* Hutch.,  *Pinanga baviensis* Becc., *Nephelium topengii* (Merr.) H. S. Lo.,  *Blastus cochinchinensis* Lour., *Ardisia virens* Kurz.,  *Beilschmiedia tungfangensis* S. K. Lee & L. F. Lau., *Gironniera subaequalis* Planch.,  *Ardisia quinquegona* Blume., *Neolitsea ovatifolia* Yen C. Yang & P. H. Huang.,  *Xanthophyllum hainanense* Hu., *Symplocos hainanensis* Merr. & Chun ex H. L. Li.,  *Platea latifolia* Blume., *Lasianthus trichophlebus* var. *latifolius* (Miq.) H. Zhu.,  *Aidia canthioides*(Champ. ex Benth.) Masam.,  *Dasymaschalon rostratum* var. *glaucum* (Merr. & Chun) Ban.,  *Symplocos adenophylla* Wall., *Memecylon ligustrifolium* Champ., |

**TABLE S2.** Model parameters of different models (Significance level:**p* < 0.05, ** *p* < 0.01, *** *p* < 0.001).

| Spatial grains | Models | R^2^ | AIC | Moran I statistic | Moran I statistic standard deviate |
| --- | --- | --- | --- | --- | --- |
| 20m×20m | OLS | 0.11 | 4107.32 | 0.18 | 14.68*** |
|  | SEM | 0.22 | 3965.45 | -0.01 | -1.20 |
| 40m×40m | OLS | 0.24 | 984.11 | 0.21 | 8.42*** |
|  | SEM | 0.36 | 935.73 | -0.01 | -0.36 |
| 60m×60m | OLS | 0.49 | 370.99 | 0.10 | 2.79** |
|  | SEM | 0.52 | 365.08 | 0.01 | 0.36 |
| 80m×80m | OLS | 0.29 | 233.85 | 0.06 | 1.46* |
|  | SEM | 0.36 | 233.80 | 0.01 | 0.43 |
| 100m×100m | OLS | 0.27 | 172.28 | 0.05 | 1.09 |
|  | SEM | 0.33 | 173.49 | -0.00 | 0.27 |


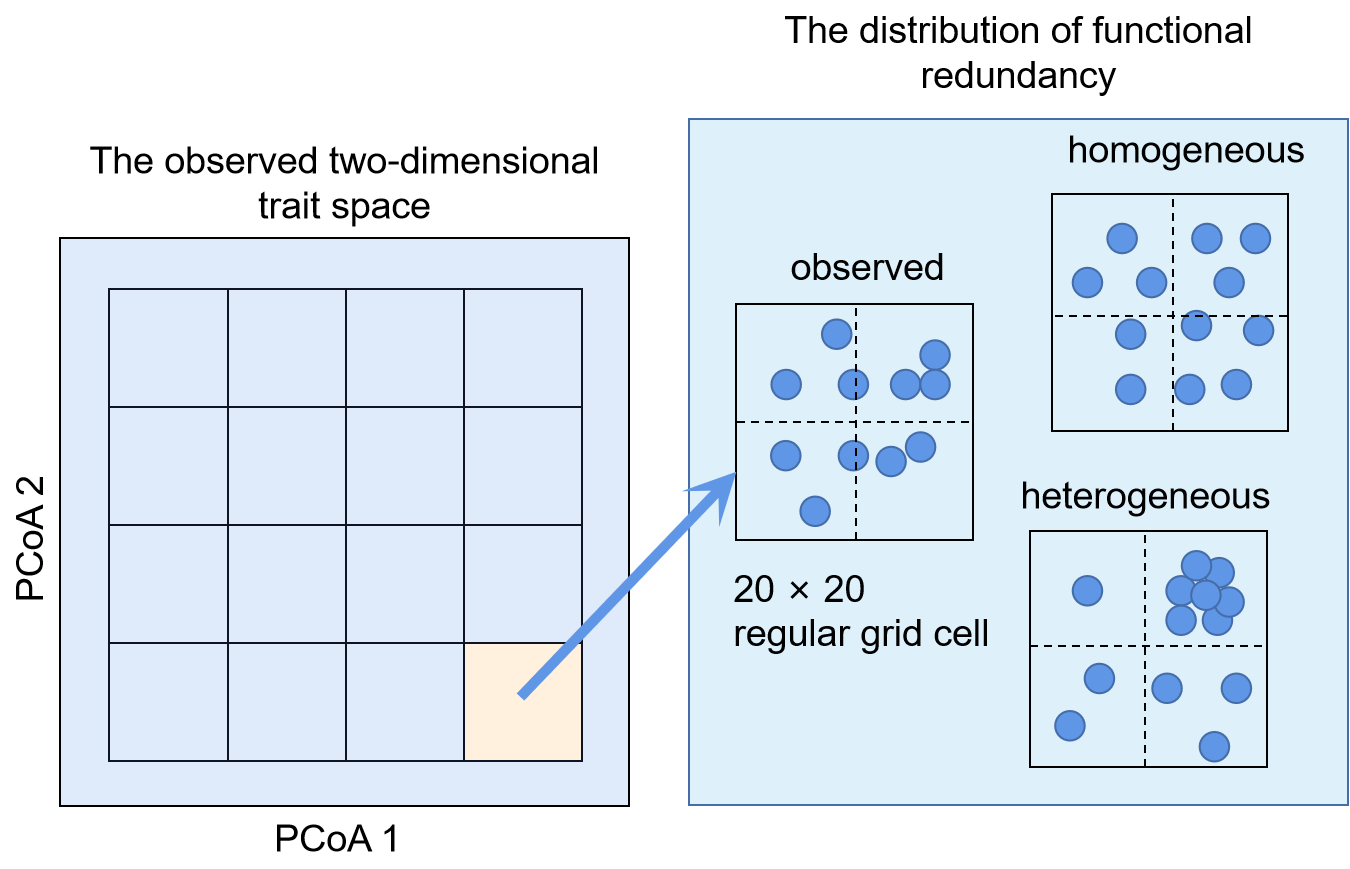


**FIGURE S1.** Trait spaces (PCoA biplots) of the 2 pseudo-communities and the observed community. Blue dots represent species.


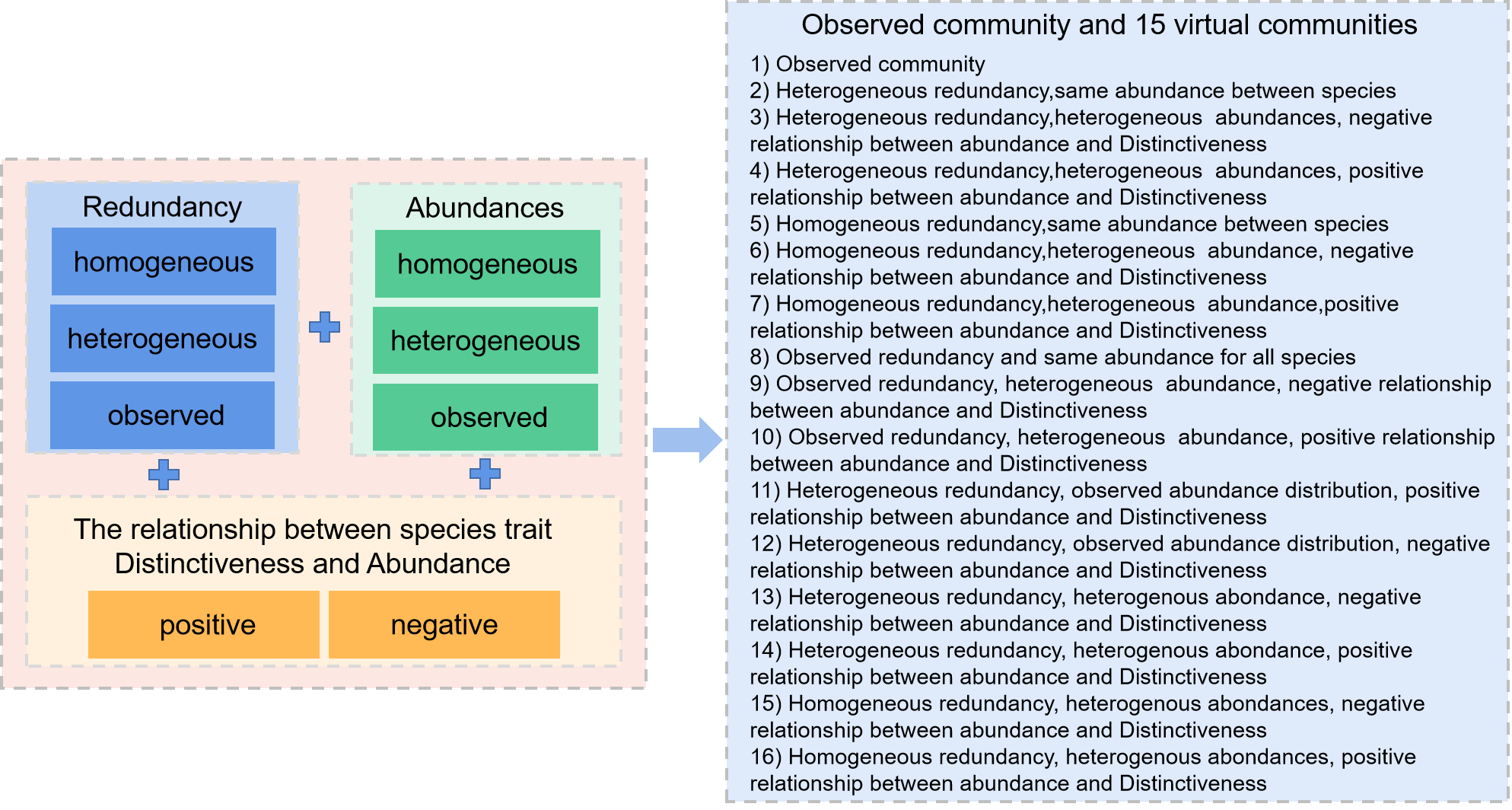


**FIGURE S2.** Observed community and 15 virtual communities (cited from (Arnaud et al., 2022)).


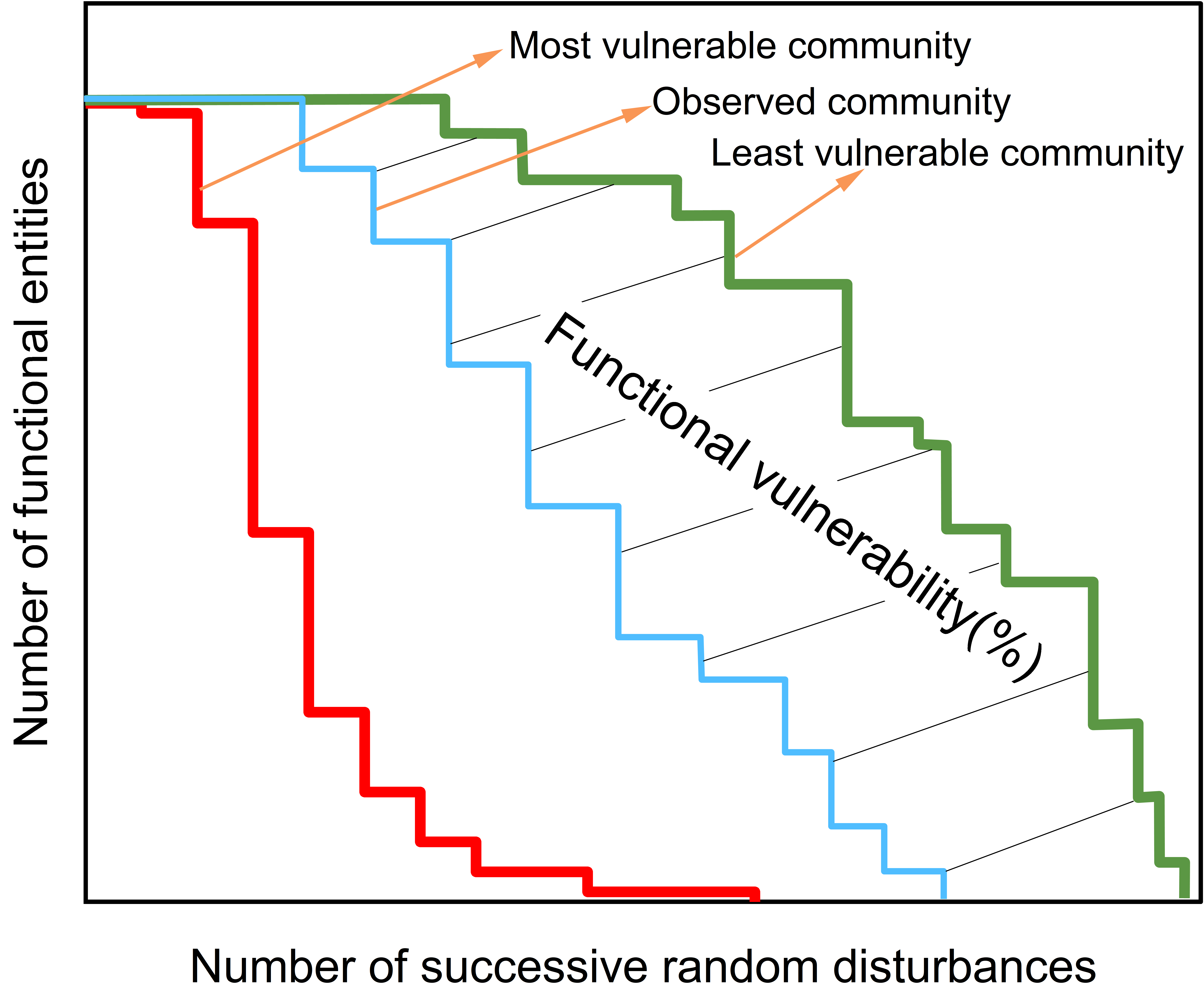


**FIGURE S3.** Conceptual figure showing the rarefaction curves of the observed target community and its associated virtual communities. Only the most vulnerable communities, the least vulnerable communities and observed community are shown here, with the remaining virtual communities being between the least and most vulnerable communities.


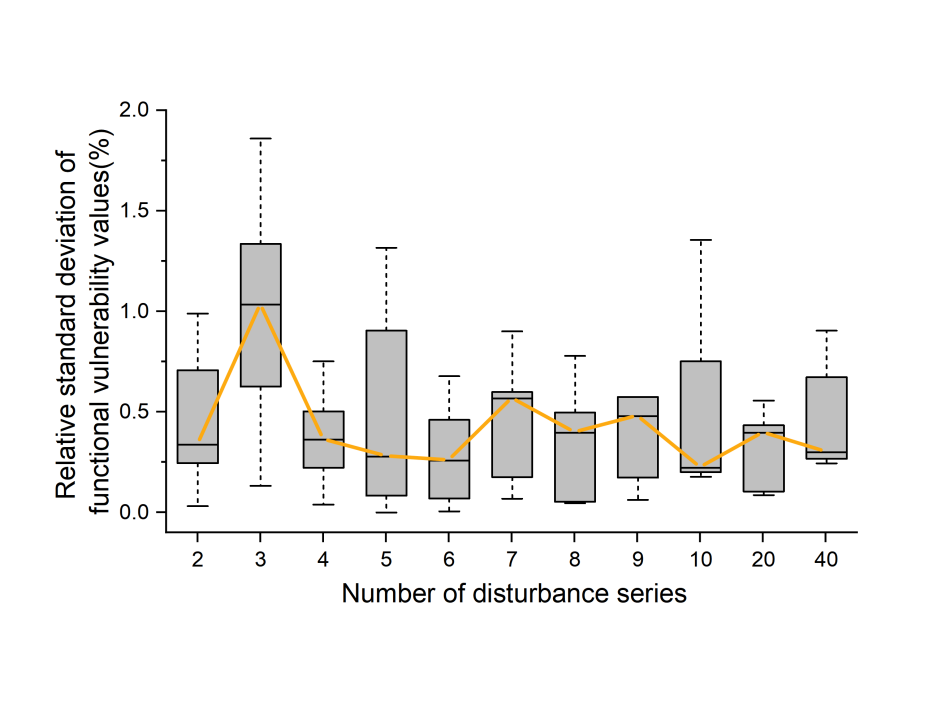


**FIGURE S4.** Sensitivity of functional vulnerability values to the number of disturbance series applied on communities. Boxes are defines by lower and upper box boundaries 25th and 75th percentiles, respectively, median is defined by the line inside box. One observed community was randomly selected and repeated 99 times with different numbers of the number of disturbance series.


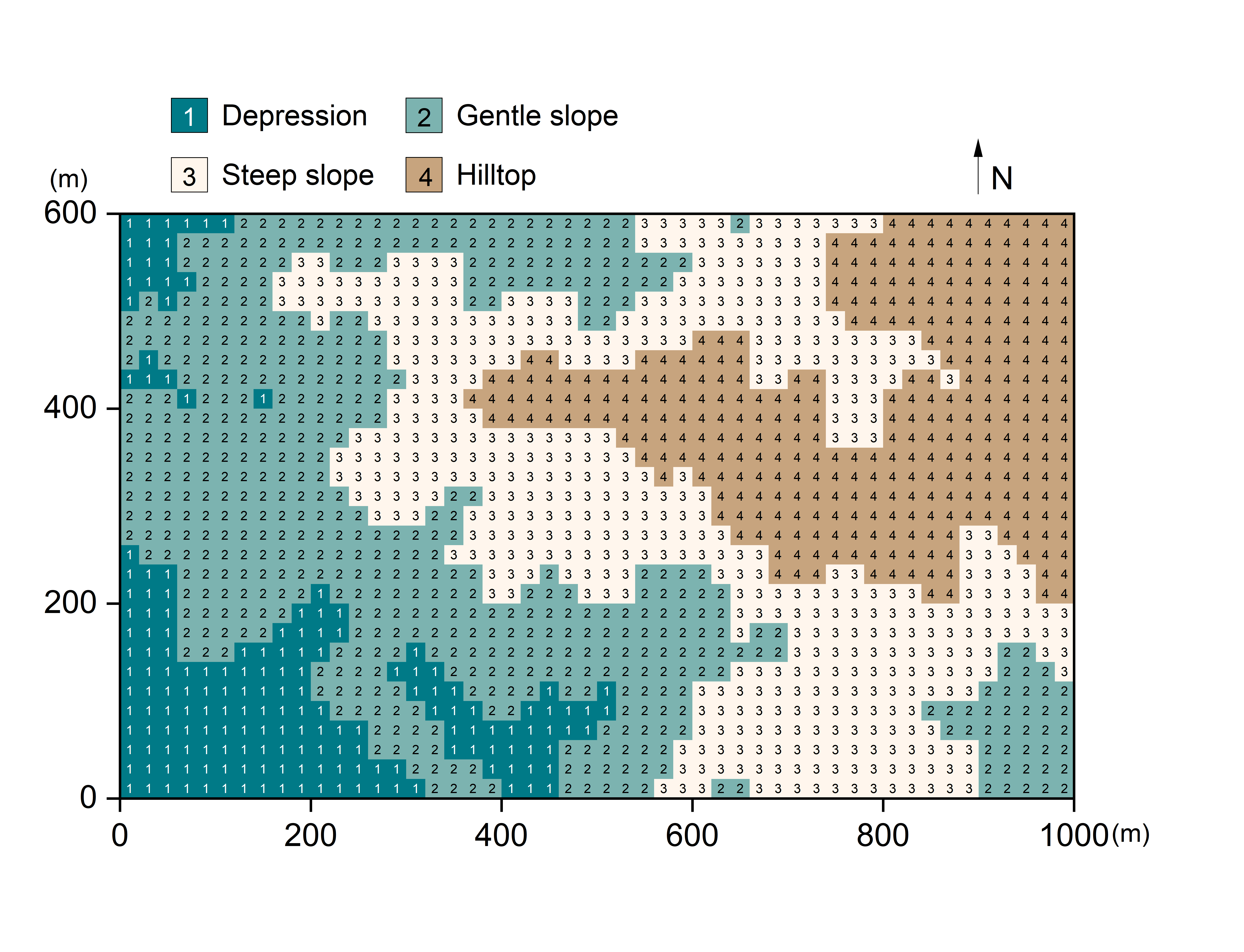


**FIGURE S5.** Clustering analysis of the topographical parameters of the 60-ha dynamics plot.


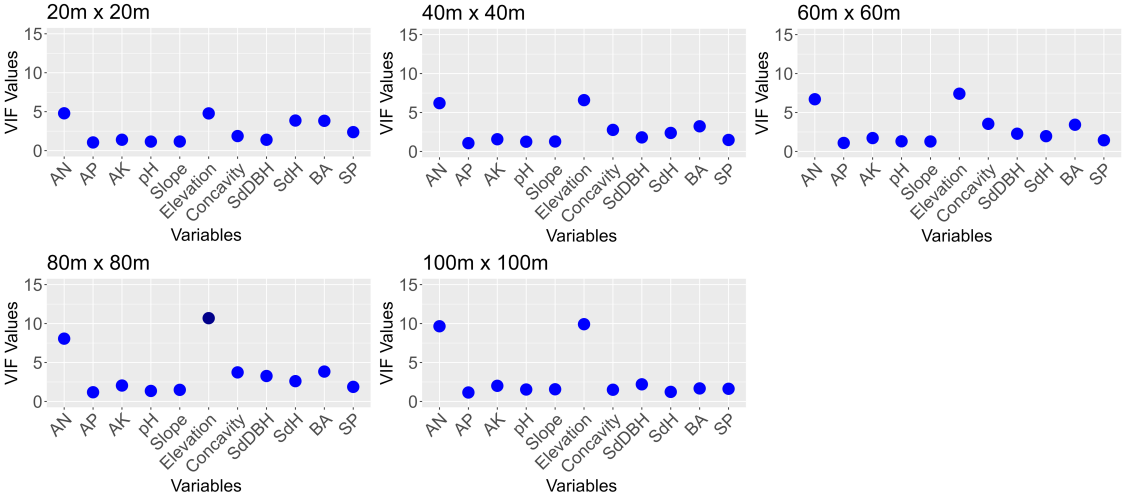


**FIGURE S6.** The variance inflation factor (VIF) used to test the multicollinearity of environmental predictors.


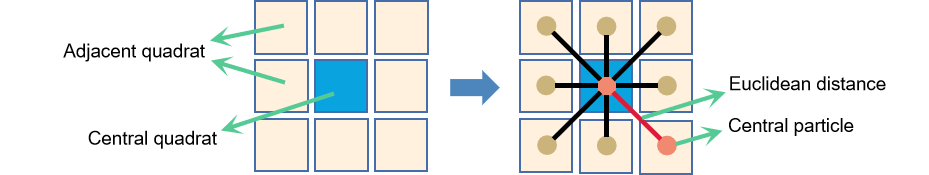


**FIGURE S7.** The method of queen contiguity was adopt to build spatial weight matrix.

**References**

Anselin, L., and Getis, A., 1992. Spatial statistical analysis and geographic information systems. The Annals of Regional Science **26**:19-33.

Arnaud, A., Conor, W., Anthony, M., Eric, G., Camille, A., C., A.A., Matthew, M., Anik, B.A., L., G.A., Mark, T., Laurent, V., Kristin, K., Kathleen, K.R., Maria, B., Jerry, T., Aurèle, T., Cyrille, V., Nicolas, M., Wilfried, T., and David, M., 2022. A functional vulnerability framework for biodiversity conservation. Nature Communications **13**:4774.

Hanbat, J., Yanli, L., and Lung-fei, L., 2023. Estimation of spatial autoregressive models for origin–destination flows: A partial likelihood approach. Economics Letters **229**:111202.
